# Preoperative Embolization With Fused CT Angiography and Tractography Facilitates Safe Resection of a Spetzler-Martin Grade IV Arteriovenous Malformation

**DOI:** 10.7759/cureus.20657

**Published:** 2021-12-24

**Authors:** Jonathan Weyhenmeyer, Josue D Ordaz, Aaron Cohen Gadol, Mitesh Shah

**Affiliations:** 1 Department of Neurological Surgery, Indiana University School of Medicine, Indianapolis, USA

**Keywords:** eloquent cortex, corticospinal tract, arteriovenous malformation, multimodal imaging, preoperative embolization

## Abstract

Brain arteriovenous malformations (BAVMs) are high-flow vascular lesions that have a propensity to rupture resulting in high rates of morbidity and mortality. Microsurgical resection of BAVMs is the standard of care for high-risk, resectable lesions. Multiple imaging modalities aid in the surgical planning and resection of high-grade BAVMs, but all have hidden variables that would prove useful if available. We present a 20-year-old male with a ruptured BAVM with concern for the involvement of the corticospinal tract (CST) and basal ganglia. We describe the melding of computed tomography angiography (CTA) and diffusion tensor imaging (DTI) in addition to preoperative embolization to aid in the planning and resection of a lesion close to eloquent structures. Post-operative CTA and DTI showed a total resection of the lesion with retained CST white matter tracts, and the patient retained the functional ability of the contralateral limbs. The combination of CTA, brain DTI, and preoperative embolization provides a framework to improve the safety of resection of BAVMs that occur near eloquent brain networks.

## Introduction

Brain arteriovenous malformations (BAVMs) are classically defined as congenital or developmental lesions consisting of arteries that drain directly into veins without an intervening capillary network [1]. They are considered to be high-flow lesions that are prone to rupture, leading to significant morbidity and mortality. The risk of BAVM rupture has historically been estimated at 2-4% per year [2,3]. The risk of hemorrhage is especially concerning because the associated mortality rate may be as high as 30% with even higher morbidity rates in survivors [3,4].

BAVMs are identified with the assistance of digital subtraction angiography (DSA) and computed tomography angiography (CTA). DSA has been the “gold standard” for intracranial vascular imaging due to its high temporal and spatial resolution. However, it provides little information in regard to surrounding structures [5]. CTA provides high spatial resolution but poor soft-tissue contrast, making the delineation of nearby brain structures challenging. With the advent of magnetic resonance imaging (MRI), the localization and relation of the lesions to eloquent brain areas has become germane [6]. However, the effects of BAVM lesions on the reorganization of involved and nearby cortical and white matter networks remain elusive based on MRI alone [7]. Functional MRI (fMRI) has provided additional information regarding the spatial relation of BAVM lesions to functional tissue based on blood oxygen level gradients obtained during task performance. Unfortunately, spatial and temporal resolution in fMRI is poor because of the underlying physical and physiologic limitations of the modality [8]. Ultimately, fMRI has not been proven to be helpful in the resection of eloquent BAVMs [9]. More recently, diffusion tensor imaging (DTI) MRI has been used to identify white matter tracts (WMTs) and their relation to BAVMs based on the anisotropic diffusion [10]. White matter tractography provides a useful roadmap for avoiding eloquent WMTs both on the approach to and during dissection around the lesion [11]. Hypothetically, DTI could provide a framework in which the traditional arteriovenous malformation (AVM) grading scales of Spetzler-Martin (SM) and Lawton-Young are augmented with an improved understanding of anatomical networks [12,13].

Each of the aforementioned imaging modalities provides useful information in the planning and surgical removal of BAVMs arising in eloquent areas. However, taken individually, each has hidden parameters that would be helpful in the planning for microsurgical resection of BAVMs in eloquent areas. To mitigate these limitations, studies have used DTI combined with T1-weighted MRI with contrast to safely resect rolandic BAVMs [14,15]. Newton et al. demonstrated 100% obliteration of the BAVMs in their series. However, all of the lesions presented were superficial and SM grades II or III. Higher grade lesions, particularly deeply seated BAVMs (e.g., basal ganglia), pose additional challenges [16]. They are more difficult to navigate in real time after opening the dura and lesion resection due to brain shifts. Identifying boundaries is paramount especially with BAVMs abutting eloquent structures such as the corticospinal tracts (CSTs) to avoid post-operative morbidity. We present multimodal image fusion of post-embolization CTA and DTI-MRI and preoperative embolization of arterial BAVM feeders to help delineate the relationship between a basal ganglia SM grade IV AVM and CST fibers in real time.

## Case presentation

**Figure 1:**
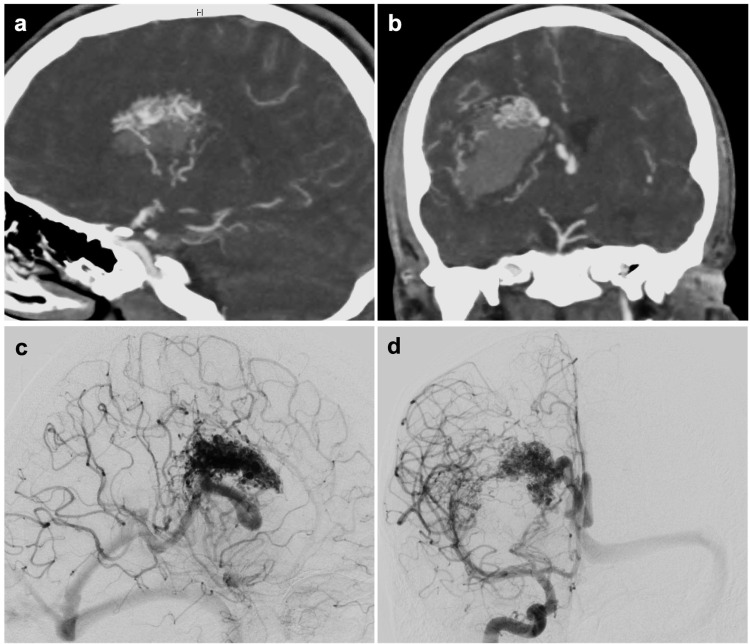
Diagnostic imaging of right frontal AVM. CT angiography of the head in the sagittal (A) and coronal (B) planes showing the right frontal AVM that is adjacent to the basal ganglia and internal capsule. (C, D) Diagnostic angiogram demonstrating AVM nidus and lenticulostriate artery feeders. AVM, arteriovenous malformation.

A 20-year-old male presented to an outside hospital after being found unconscious. The emergency department workup and CT of the head revealed a right-sided intraparenchymal hemorrhage with intraventricular extension and evolving hydrocephalus. CTA of the brain revealed a right basal ganglia arteriovenous malformation abutting the internal capsule and measuring 3.9 x 2.9 x 1.7 cm (Figures 1A, 1B). He was subsequently transferred to our facility. An external ventricular drain was placed emergently for the treatment of hydrocephalus. He was hemiplegic on the left and had a Glasgow Coma Scale score of 13 without signs of impending herniation. Digital subtraction angiography revealed a Spetzler-Martin grade IV AVM (Figures 1C, 1D). Based on his presentation and clinical status, the decision was made to medically stabilize the patient with a plan for resection four to six weeks after the initial hemorrhage.

**Figure 2:**
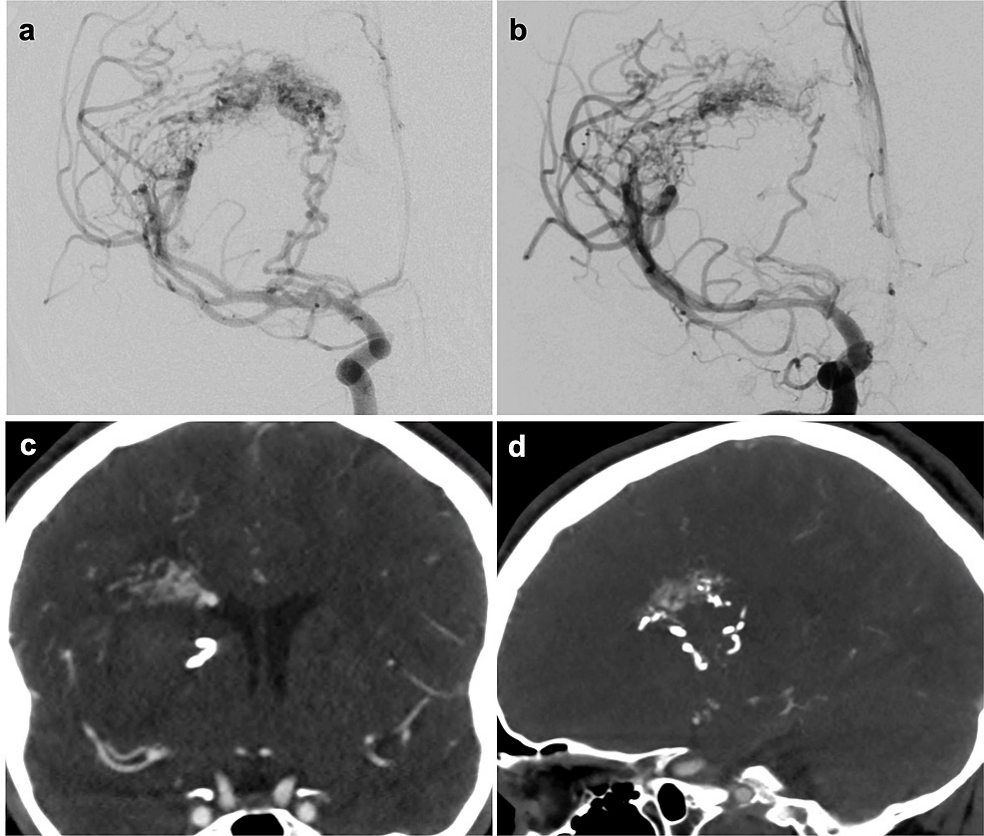
Angiogram and CTA post-embolization. Right ICA angiogram demonstrating (A) pre- and (B) post-embolization of two lenticulostriate artery feeders. Coronal (C) and sagittal (D) CTA demonstrating embolized AVM arterial feeders. CTA, computed tomography angiography; ICA, internal carotid artery; AVM, arteriovenous malformation.

**Figure 3:**
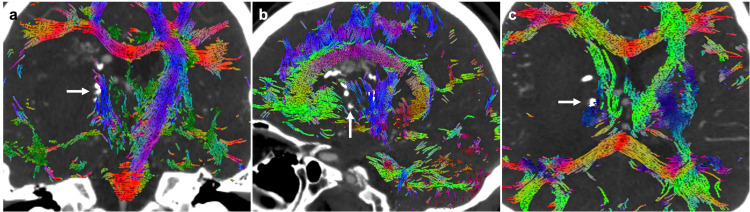
Post-embolization CTA and DTI fusion. Post-embolization CTA in the coronal (A), sagittal (B), and axial (C) slices with DTI tractography overlaid. The CST is shown to wrap posteromedial to the hemorrhage and embolized AVM feeders (arrow). CTA, computed tomography angiography; DTI, diffusion tensor imaging; CST, corticospinal tract; AVM, arteriovenous malformation.

The patient was scheduled for an elective AVM resection two months after the initial presentation and discussion in a multidisciplinary neurovascular conference. Initially, after admission to the hospital for elective surgery, an MRI with diffusion tensor imaging (DTI) was performed. Next, endovascular embolization was attempted to help facilitate resection and establish the medial border of the BAVM. Only two out of the three lateral lenticulostriate artery feeders were successfully embolized (Figures 2A, 2B). An additional CTA of the head was performed post-embolization, which demonstrated the embolized arterial feeders (Figures 2C, 2D). On the date of surgery, the previously obtained MRI with DTI and post-embolization CTA were fused utilizing a proprietary algorithm (Synaptive Medical©, Toronto, Canada; affine transformation) (Figures 3A-3C). The fused images provided clear information regarding the trajectory of the corticospinal tracts as they wrapped around the posterior and medial aspect of the AVM and hemorrhage cavity. In addition, the embolization material was evident at the posterior aspect of the AVM (Figure 3, arrows) and abutted the CST. This provided an ideal landmark that indicated that CST tracts were running adjacently. During the resection, the embolized material was clearly felt with the bipolar forceps and suction instruments. This provided a stopping point for the posterior and medial aspect of the resection and facilitated safe removal of the nidus.

**Figure 4:**
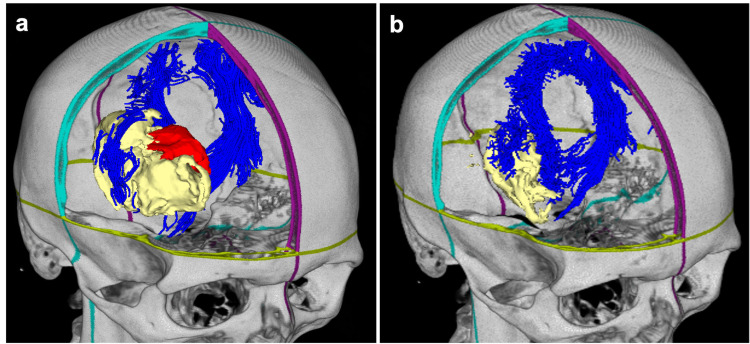
Pre- and post-operative 3-dimensional reconstruction. (A) Pre-operative AVM (red), hemorrhage (yellow), and CST (blue) reconstruction clearly delineating the spatial relationships. (B) Post-operative reconstruction displaying the relationship of the CST to the resection cavity. The CST is shown to wrap medially and laterally around the resection cavity. CST, corticospinal tract; AVM, arteriovenous malformation.

**Figure 5:**
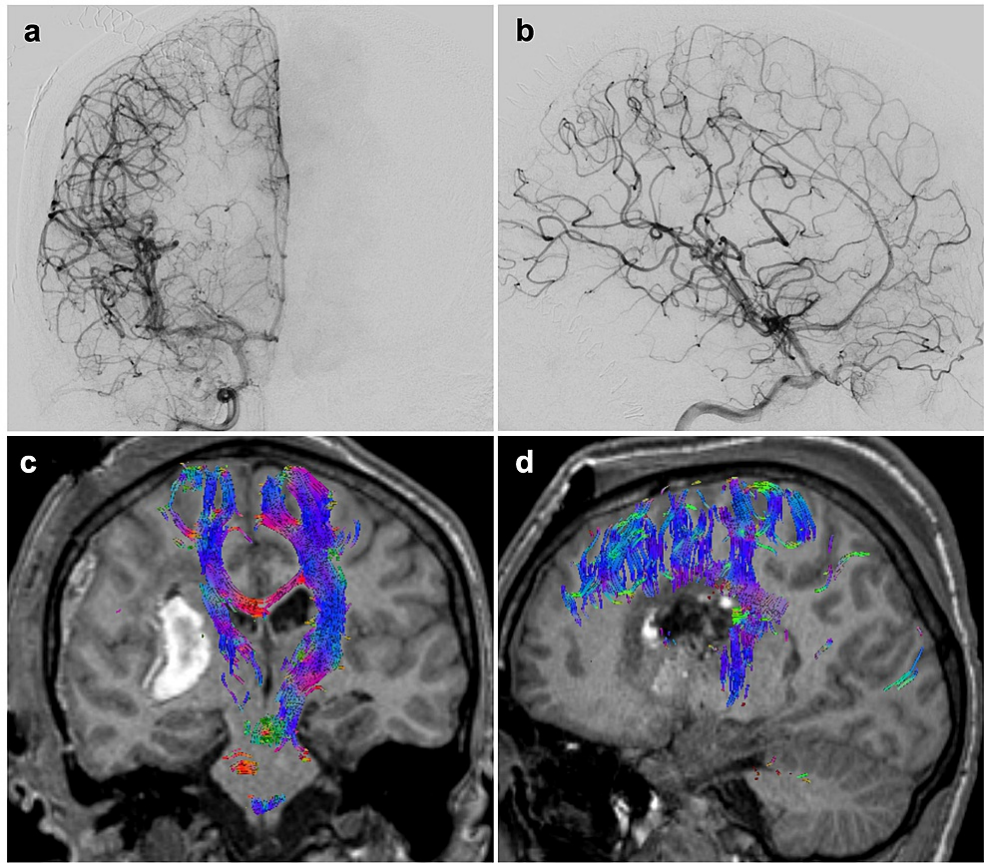
Post-operative MRI with DTI and angiogram. (A, B) Post-operative MRI with DTI sequence in the coronal (A) and sagittal planes (B) showing preservation of the CST. (C, D) Post-operative lateral and AP angiography displaying complete resection of the AVM nidus. DTI, diffusion tensor imaging; CST, corticospinal tract; AVM, arteriovenous malformation; AP, anteroposterior.

A pre- and post-operative three-dimensional reconstruction is provided, which displays the CST wrapping medially and laterally around the posterior aspect of the resection cavity without apparent damage to the CST (Figure 4). A post-operative angiogram and MRI with DTI sequence were performed and confirmed gross total resection of the BAVM and intact CST (Figure 5). The patient gradually improved postoperatively. During a three-month clinic follow-up, he was able to walk with the assistance of an ankle orthosis. He continued to have mild spasticity on the left side, which was being managed medically and continued to improve. He had good use of his left arm and could squeeze and open his hand.

## Discussion

The Spetzler-Martin AVM grading scale has provided a useful metric for surgical morbidity and mortality based on imaging characteristics since it was initially described in 1986 [12]. AVMs with Spetzler-Martin grades I-II have been considered surgically resectable with acceptable rates of perioperative morbidity and mortality while grades IV-V have been considered too dangerous to advocate for resection. While this grading scale has stood the test of time, it utilizes crude imaging findings (e.g., anatomical location in eloquent or non-eloquent brain) in its computation. The response of cortical and subcortical networks to adjacent and intervening AVMs has not been well described. As such, it is difficult to ascertain based on MRI and CT alone whether an AVM lesion truly resides within eloquent cortical and/or subcortical networks. While functional MRI would be expected to help delineate functional from non-functional tissue near an AVM lesion, it has unfortunately not been able to do so due to the physical limitations of the modality [8]. DTI has provided new information regarding WMTs and their relation to surrounding lesions. The additional information provided by DTI may thus alter the grading of an AVM and its presumed operability.

The grading of an AVM should not be considered static over time. The plasticity of eloquent networks may serve to rearrange WMTs such that a previously unresectable AVM may become resectable. In addition, hemorrhagic events may mobilize or destroy eloquent networks, at which point microsurgical resection can be performed without causing further morbidity to the patient. In this article, a patient with a grade IV AVM in an eloquent location presented with a hemorrhagic event. The hemorrhage pushed the cortical spinal tracts and basal ganglia away from the nidus, providing a safe pathway for microsurgical resection. The use of static imaging (i.e., CTA and MRI with DTI) is critical for surgical planning. However, its reliability decreases after dura opening and intradural dissection. This is particularly true for deep lesions. To navigate the BAVM in real time and identify the medial border, preoperative embolization of the lateral lenticulostriate artery feeders was performed. The usefulness of preoperative embolization was recently studied by Catapano et al. after conducting a propensity-adjusted analysis from a double institution cohort of SM grade III AVMs [17]. They found non-embolization of intermediate-risk AVMs was associated with poorer functional outcomes and hypothesize preoperative embolization provides benefit by facilitating nidus dissection, occluding deeper, more difficult to reach arterial feeders, and improving hemostasis. The ability of preoperative embolization to delineate the borders of a deep AVM was not described, but this technique is widely used to aid in the resection of high-grade BAVMs [18].

## Conclusions

The combination of fused CTA and brain DTI imaging with preoperative embolization of arterial feeders provides a framework to improve the safety of resection of high-grade BAVMs that occur near eloquent brain networks. Preoperative embolization delineated the deep border of the BAVM in real time while the fused CTA and DTI image helped navigate around the corticospinal tract. The ability of our patient to move the corresponding side of his body and subsequently regain additional function provides evidence that his AVM was microsurgically resected with sparing of the surrounding eloquent networks.
